# Peripheral Nerve Stimulation for Chronic Foot Pain Induced by Morton Neuroma: A Novel, Personalized Approach in One Patient

**DOI:** 10.1227/neuprac.0000000000000043

**Published:** 2023-06-12

**Authors:** Brian Fabian Saway, Reese Townsend Terry, Aimee C. Weber, Nathan C. Rowland

**Affiliations:** ‡ Department of Neurosurgery, Medical University of South Carolina, Charleston, South Carolina, USA;; § Johns Hopkins University Krieger School of Arts and Sciences, Baltimore, Maryland, USA

**Keywords:** PNS, Morton neuroma, Foot pain, Functional neurosurgery

## Abstract

**BACKGROUND AND IMPORTANCE:**

Morton neuroma (MN) is a condition characterized by pain that is located within one or more intermetatarsal spaces of the forefoot. Numerous conservative measures are available for the management of mild-to-moderate MN cases. In more severe presentations, surgical interventions may be considered, including neuroma excision, cryogenic or radiofrequency ablation, and decompression. However, no standard treatments exist for occurrences of recurrent MN after surgery. Peripheral nerve stimulation is a neuromodulatory treatment that is highly effective for other neuropathic pain syndromes but remains underutilized for MN.

**CLINICAL PRESENTATION:**

Here, we present the operative technique and clinical outcome in a patient with chronic pain induced by MN who underwent bilateral implantation of peripheral nerve stimulation devices. In less than 12 months, the patient had near-complete resolution of chronic foot pain with no postoperative complications.

**DISCUSSION:**

We present the first published case and operative technique of successful peripheral nerve stimulator implantation as an effective treatment of MN.

**CONCLUSION:**

Peripheral nerve stimulator implantation is a promising and effective intervention that can be considered for refractory MN.

**ABBREVIATIONS:**
IPG: implantable pulse generator
MN: Morton neuroma
PNS: peripheral nerve stimulation.

Morton neuroma (MN) is characterized by pain in the intermetatarsal spaces of the forefoot. Pain induced by MN can occur unilaterally or bilaterally and is characterized by burning, stinging, or painful numbness.^1-6^ Chronic or persistent cases represent 10% to 19% of the population with MN and can be physically and emotionally debilitating.^1^

Peripheral nerve stimulation (PNS) has not, to date, been used to treat MN. Here, we describe the operative technique of a 78-year-old man with treatment-resistant chronic pain induced by bilateral MN that was treated with PNS.

## CLINICAL PRESENTATION

### Clinical Findings and Diagnostic Assessment

A 78-year-old male patient presented with bilateral MN and over 15 years of persistent bilateral forefoot pain. The pain was described as sharp, burning pain involving the third web space bilaterally. Criteria for diagnosis of MN included a positive +3 Mulder click test and exquisite pain on bilateral compression of the third metatarsal base.

### Timeline

Over the course of 15 years, the patient had attempted conservative management with NSAIDs, antidepressants, oral anticonvulsants, and opioid analgesic medication. He then underwent 6 rounds of chemical neurolysis injections, peripheral nerve block, and surgical intervention with bilateral synovectomies of the transverse ligaments. All these resulted in moderate, temporary relief with eventual return of symptoms.

Given that conventional management strategies had failed and local treatments had transiently modulated the patient's pain, PNS leads targeting the affected nerves was put forth as a treatment option, for which the patient consented.

### Therapeutic Intervention: Implantation of Trial Stimulators

**FIGURE 1.**
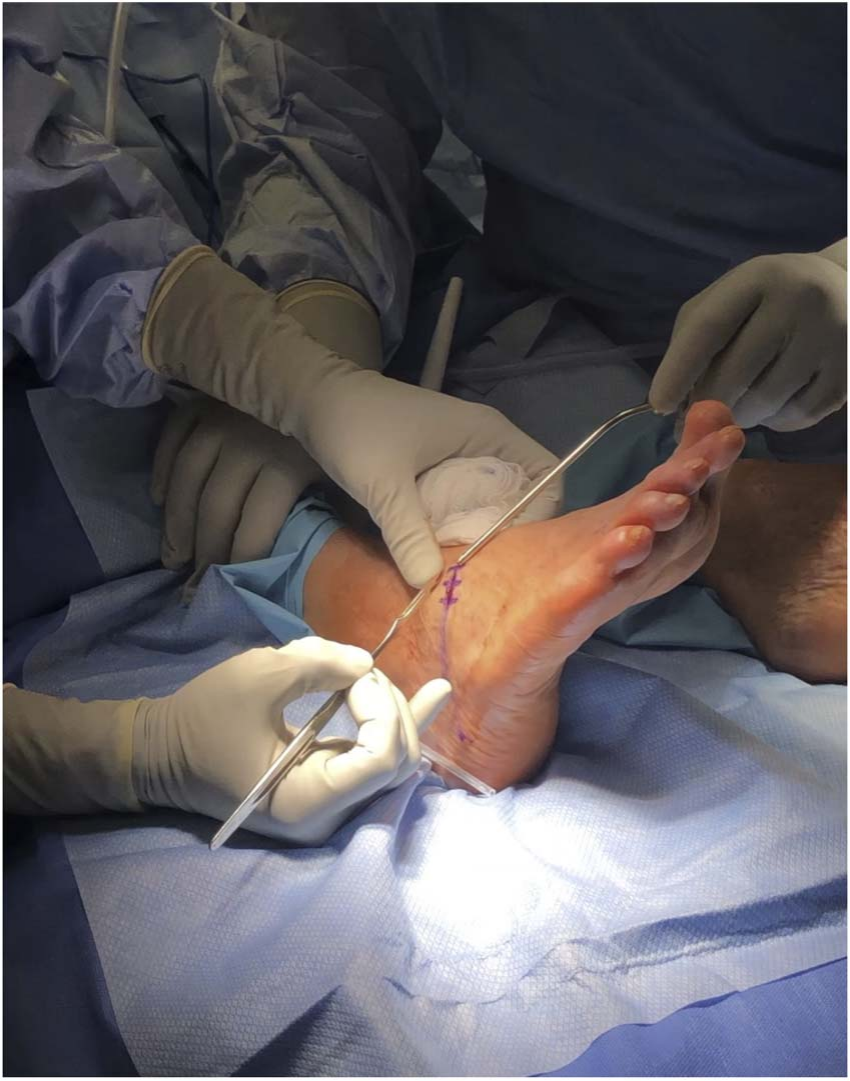
Transverse incision 5 cm proximal to distal interphalangeal joint for the trocar insertion entry point.

**FIGURE 2.**
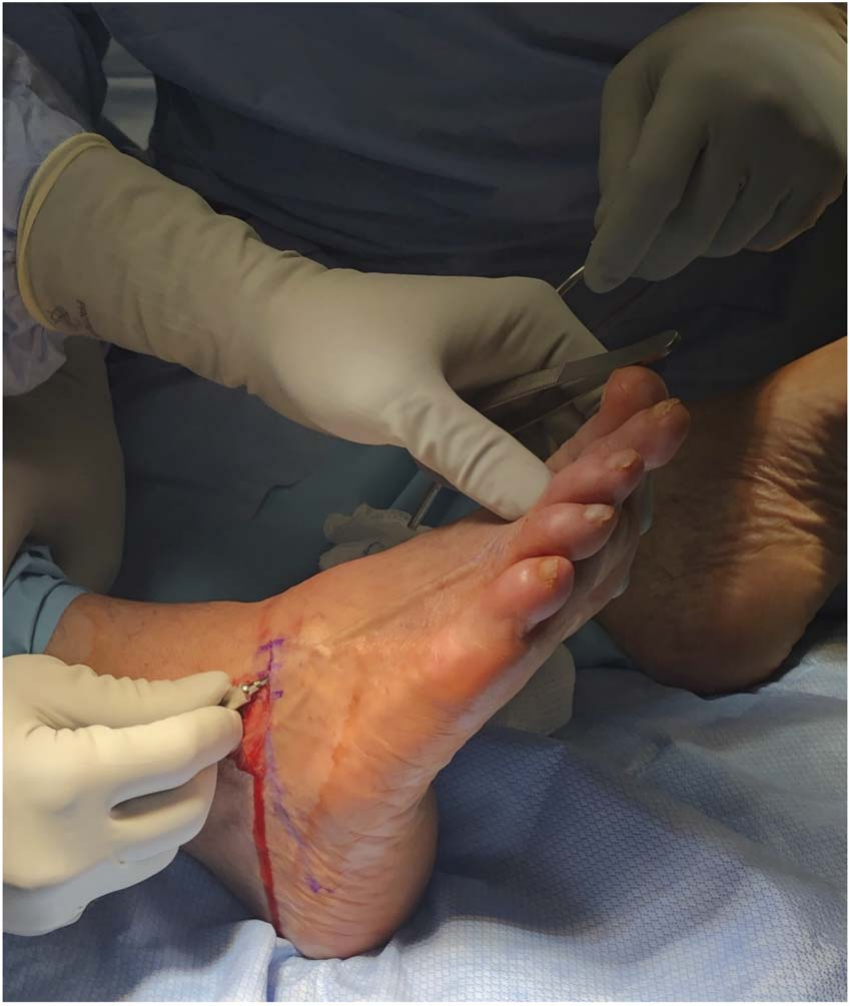
Trocar trajectory and placement in the interdigital space, best approximation of interdigital nerve location.

**FIGURE 3.**
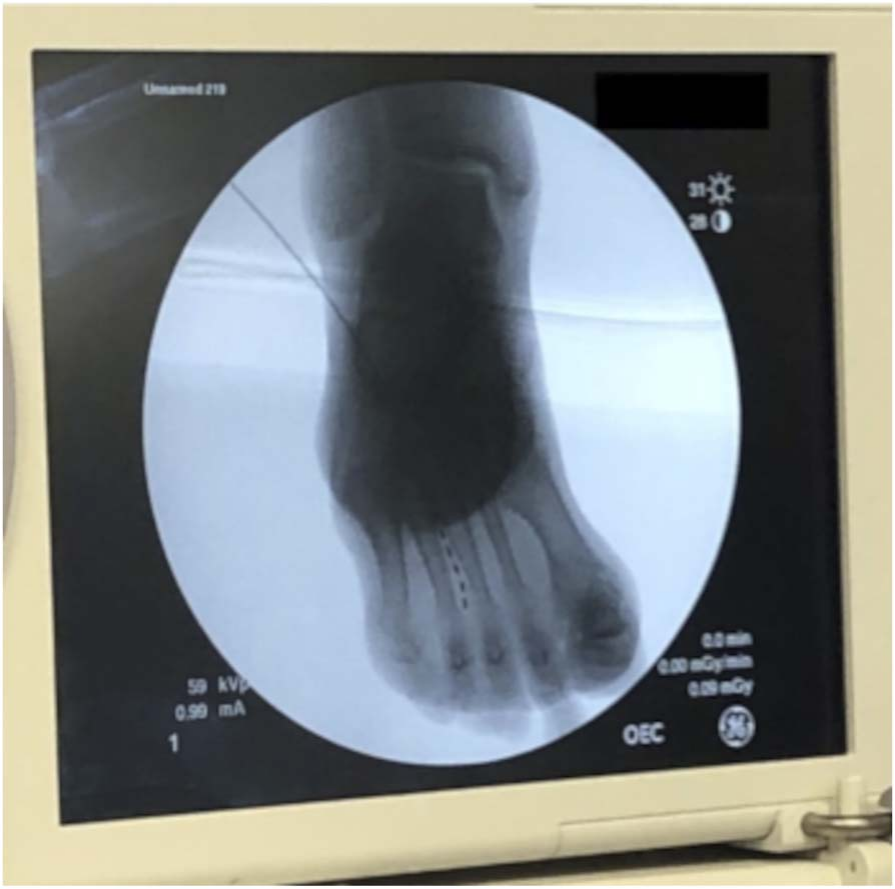
Fluoroscopic confirmation of peripheral nerve stimulation lead placement at the third web space.

**FIGURE 4.**
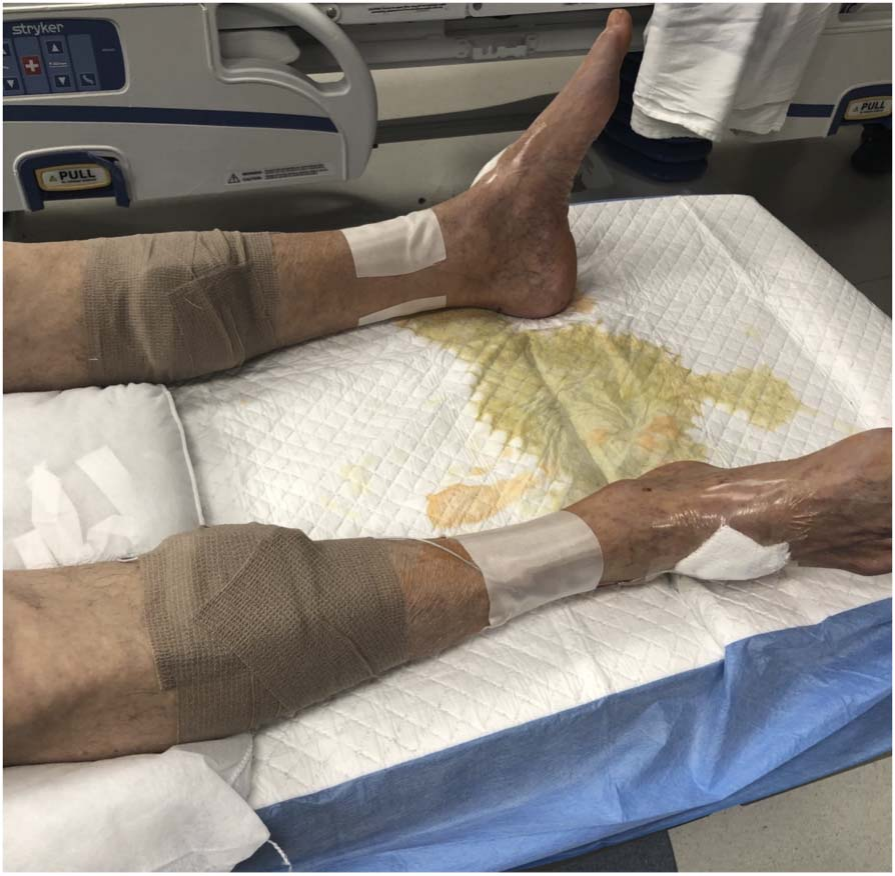
Demonstration of placement of temporary batteries along bilateral medial calves.

The patient was positioned supine on the operating table with both feet and calves sterilely draped. Beginning on the right foot, a transverse incision 5 cm proximal to the distal interphalangeal joint was made (Figure 1). This location was chosen due to the presence of a small dorsal fat pad that would later be needed to support the anchoring device for the permanent lead, while also not located too posteriorly near the tibiotalar joint such that the anchor and/or lead would be mobilized with dorsiflexion. A stylet and a straight trocar were then used to access the area overlying the interdigital nerve at the third intermetatarsal space (Figure 2). Once the target location was reached, the stylet was removed and a trial PNS lead (Model # 977D260, Medtronic) was inserted. Placement was then confirmed using fluoroscopy (Figure 3). The trocar was then removed, and the lead was anchored into the final position with a 2-0 nylon suture. The surgical procedure was then repeated for the left foot. External stimulators were secured to the patient's calves with cohesive bandage wrap (Figure 4).

After the implantation of the trial leads, the patient endorsed that he had bilateral, near-complete pain relief in his feet when the stimulators were turned on. As such, the patient was approved to have the permanent stimulators implanted.

### Implantation of Permanent Stimulators

For the permanent procedure, the nylon sutures were removed and the trial leads were replaced with permanent leads (Model # 977A260, Medtronic) along with anchoring devices at the dorsal fat pad described previously. Next, a 5-cm vertical incision was made on the lateral calf, which was used to form a pocket for the implantable pulse generator (IPG) (Model # 97715, Medtronic). A tunneling tool was then used to connect the foot incision to the calf incision to pass the terminal ends of the PNS leads to the sural pocket. The lead terminals were connected to the IPG which was then inserted into the pocket and anchored with 2-0 silk sutures and the incisions were closed with an absorbable suture.

### Follow-up and Outcomes

**FIGURE 5.**
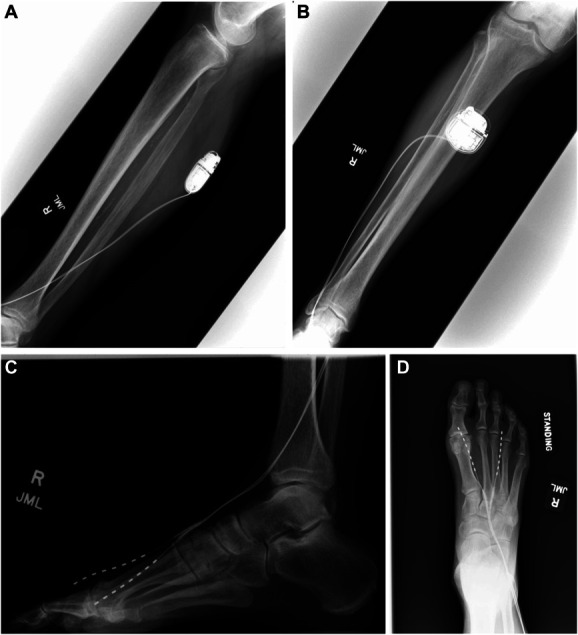
**A**-**D**, Postoperative x-rays of the patient's lower extremity demonstrating positioning of the lead and implantable pulse generator as well as the path of the tunneled lead.

No postoperative complications occurred. Postoperative x-rays detailed stable hardware placement without lead migration (shown in Figure 5A-5D). Initial visual analog scale (VAS) pain rating was 6 of 10, which decreased on each subsequent visit with sequential reprogramming. At the 10-week postoperative visit, the patient reported that the current settings controlled his pain effectively with a consistent rating of 1 of 10 on the VAS. The patient did note mild paresthesias in the lower legs that were not bothersome. This clinical efficacy was consistent at the 14-month follow-up after the procedure.

## DISCUSSION

MN is a thickening of the interdigital nerves that pass below the distal metatarsal transverse ligament. MN can progress over time and lead to significant declines in quality of life.^2-4,6,7^

Initial treatment strategies for MN are behavioral, including avoidance of narrow shoes, followed by conservative management with oral NSAIDS and anticonvulsants, local injection, radiofrequency ablation, and cryotherapy.^1,4,7-10^ Efficacy for noninvasive MN treatment measures are historically on average approximately 50%.^7,10^ The next line of treatment includes surgical excision, although this strategy risks significant discomfort for patients with chronic cases and have recurrence rates that are not negligible. A study by Johnson and colleagues analyzed 37 patients with recurrent neuroma after excision procedures and found that 21% of participants had signs of stump neuroma and 46% of participants showed signs of both existing primary neuroma and stump neuroma.^4,11,12^ Beskin and Baxter^13^ reported outcomes from 30 patients with MN who underwent surgical excision and found that less than 50% experienced complete relief and over 58% continued to experience high levels of pain.

Despite the long history of PNS as an effective treatment of pain, there are no documented cases of PNS being applied for MN.^14,15^ In this current report, a 78-year-old man underwent successful bilateral peripheral nerve stimulation along the third common plantar digital nerve to relieve chronic forefoot pain associated with MN. Lead implantation on either side of the painful area allowed for the use of bipolar montages to create a spreading “field effect” in between the leads. It is important to note that other neuromodulatory interventions, such as spinal cord stimulation, for chronic foot pains have been reported in the literature with promising clinical efficacy.^16^ This treatment option was discussed with the patient as a possible next step if the PNS trial was unsuccessful.

A notable aspect of the operation is the placement of the generator over the lateral calf bilaterally. Because this was an unprecedented procedure, it was necessary to devise a location that did not lead to patient discomfort, did not prevent proper wound healing, and was not likely to cause lead migration or fracture based on the position in a highly dynamic region. It was decided to place the generators in a pocket in the lateral calf, allowing the leads to travel down a short distance to reach the metatarsal area. After the procedure, the patient did report feeling paresthesias in his lower legs. It is possible that the onset of this sensation may be attributed to the tunneling portion of the procedure that may have caused injury to the various sensory nerves of the anterior, lower leg such as the superficial peroneal nerve.

Although this is a single case of successful bilateral implantation of PNS in the metatarsal space for the treatment of MN, larger trials are needed to validate the efficacy of this technique.

## CONCLUSION

We present a case demonstrating that PNS can successfully be used to treat chronic, treatment-resistant MN.
